# Plants and Plant-Derived Molecules as Natural Immunomodulators

**DOI:** 10.1155/2023/7711297

**Published:** 2023-06-05

**Authors:** Meseret Zebeaman, Mesfin Getachew Tadesse, Rakesh Kumar Bachheti, Archana Bachheti, Rahel Gebeyhu, Kundan Kumar Chaubey

**Affiliations:** ^1^Center of Excellence in Nanotechnology, P.O. Box 16417, Addis Ababa, Ethiopia; ^2^Department of Industrial Chemistry, Addis Ababa Science and Technology University, College of Applied Science, P.O. Box 16417, Addis Ababa, Ethiopia; ^3^Centre of Excellence in Biotechnology and Bioprocess, P.O. Box 16417, Addis Ababa, Ethiopia; ^4^Department of Environment Science, Graphic Era University, Dehradun, 248002 Uttarakhand, India; ^5^Microbiology Department, Armauer Hansen Research Institute, Addis Ababa, Ethiopia; ^6^Division of Research and Innovation, Uttaranchal University, Arcadia Grant, P.O. Chandanwari, Premnagar, Dehradun, Uttarakhand 248007, India

## Abstract

*Background*. Nowadays, the immunomodulatory properties of plants have been studied extensively with greater interest due to increasing awareness and combating the severity of immunomodulatory diseases. *Scope and Approach*. This paper highlights the efficacy of the available literature evidence on natural immunomodulators of plant origin and synthetic ones. In addition, several aspects of plants and their phytoconstituents responsible for immunomodulation have been discussed. Moreover, this review also discusses the mechanism involved in immunomodulation. *Key Findings*. One hundred fifty medicinal immunomodulatory plants are currently identified to find novel immunomodulatory drugs. Of these plants, the plant family *Asteraceae* also takes the first rank by offering 18 plant species (12%). Similarly of the plants studied so far, 40% belong to the *Asteraceae* family. *Echinacea purpurea* of this family is most known for its immunostimulating activity. The most prominent immune-active bioactive molecules are polyphenols, terpenoids, and alkaloids. Also, eight plant bioactive immunomodulators were checked for clinical trials and found in the market. These are six immunosuppressants, resveratrol, epigallocatechin-3-gallate, quercetin, colchicine, capsaicin, and andrographolide, and two immunostimulants, curcumin and genistein. Nowadays, there are a lot of polyherbal traditional medicinal products sold in the market and claimed to their immunomodulators. However, much work is still needed to find more active immunomodulatory agents. The mechanism by which immunomodulatory medicinal plant exert their effect is through the induction of cytokines and phagocyte cells and the inhibition of iNOS, PGE, and COX-2 synthesis.

## 1. Introduction

Nowadays, in the 21st century, the world health sector is facing different problems. Among the problems faced by the scientific communities are antibiotic resistance [1], antiviral resistance, and anticancer resistance [2], side effects of commercial drugs [3], undeveloped drug delivery mechanism, treatment of cardiovascular disease, metabolic disorder, COVID-19, inflammation, and neurological disorder. The scientific community forecasts that due to only antibacterial resistance, by 2050, ten million people's death is expected [4]. Twenty-two million people's death is also expected by 2030 due to cardiovascular disease [5, 6].

However, despite this burden, the scientific community is also looking for a solution by relying on traditional medical practice knowledge. One of the scientific community's solutions is that disease prevention needs much effort as working on the curing agent [7]. Among the prevention mechanism, which is also stated in the traditional health care system of different parts of the world, enhancing the human immune system is one solution to reduce the increasing incidence of diseases and deaths [8]. Immunotherapies using plant-sourced phytochemicals are now getting attraction to combat the spread of cancer, autoimmune disease, and infection [9].

The second intersection point of agreement between the scientific community and traditional medicinal plant practitioners is that it is good to study and use traditional medicine called natural products since they are multivalent component agents than monovalent commercial drugs [10, 11]. Medicinal plants are the best reservoirs of bioactive compounds. The are less expensive and easily available and have less side effects which make them suitable for producing drugs [12].

According to various traditional medical systems around the world, such as the Indian Ayurvedic system, it is beneficial to increase body resistance. Even in Ayurvedic, one chapter is called “Rasayana” which means plant drug, herbal medicine, traditional, or natural medicine reputed to increase body resistance [13]. Nowadays, it is called immunity-enhancing medicinal plants. These plants, listed under the “Rasayana” chapter, prevent diseases like aging, cancer, diabetes, autoimmune disease (like rheumatoid arthritis), and Parkinson's disease. Like the Indian Ayurvedic, there is a traditional health care system worldwide like those found in Egypt, China, Ethiopia, Western, Kampo (Japan), and Greco-Arab or Unani-Tibb (South Asia) [13].

Despite the traditional health care system, the scientific community clinically classifies immunity enhancement method into three. The first and second are called immunization and taking probiotic food, while the third is called vaccination. Immunization is self-immunity development after exposure to disease. Probiotic foods, for example, fermented foods, cause the development of useful bacteria in the guts and hence improve the human body immune system. For instance, a review study done by [14] indicates that these foods' immunomodulatory activity is because of their rich content of antioxidant and lactic acid-producing bacteria. Besides this, taking appropriate food is also considered as one means of immunity-enhancing methods. For example, a study done by [15] indicates that bell pepper and carrot have a phytochemical called rhamnogalacturonan-I (RG-I) that stimulates innate immune system in our body. However, in general, there are eight immune-enhancing methods for a healthy man to follow [16].

Scientifically, immunity systems are divided into innate immunity and adaptive immunity. Generally, the type of natural medicine and synthetic drugs as immunomodulators, which will be discussed later, is divided into three: immunomodulators, immunosuppressant, and immunoadjuvant [17, 18].

The Medical Herbalism book written by Hoffman in 2003 [19] and the book by Pal and Nayak in 2021 [20] classify the phytochemical immunomodulators into two: low and high molecular weight compounds. Similarly, there are two modes of action by which immunomodulatory compounds exert their effect, namely, specific and nonspecific [19, 20].

**Figure 1:**
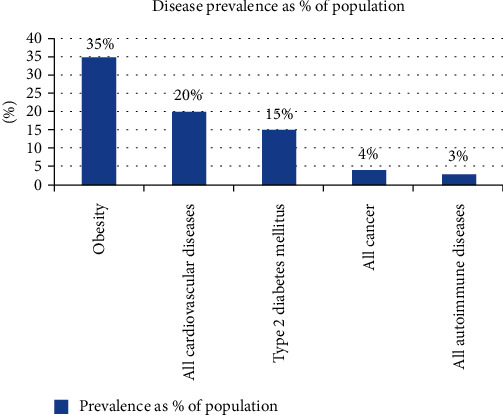
Prevalence of disease report by 2022 in the USA. Adopted from the immunology department of Johns Hopkins University website in 2022.

Taking immunomodulators is mostly used to boost the immune mechanism in immunocompromised patients and suppress the immune system of those who by autoimmune disease are affected. According to Johns Hopkins University's pathology department website information in the USA, autoimmune diseases affect 3% of the USA population (Figure 1) [21].

In addition to autoimmune diseases, immunodeficiency diseases of the first type, primary immunodeficiency disease (PIDD), also affect the world population. Genetic defects of antibodies cause PIDD, and until now, around 130 PIDD types are identified [22]. A systematic global review of PIDD registries from January 1981 to June 2020 published and unpublished studies indicate that 13852 (13.2%) of all registered 104614 PIDD patient molecular defects in genes are identified to cause PIDD. From the total registered PID, approximately 10590 additional PID patients are registered from Asia and Africa [23].

This review highlights the research done on immunomodulators of plant origin and synthetic agents. As introduction, for example, a study done by [24] indicates elderberry plant extract called eldosamb® has the ability to reduce the production of cytokines like TNF-*α* and IFN-*γ* and hence cause the initiation of Th2-helper cell, which means adaptive immune system. This product is now available in the market. However, this review also highlights mostly unmarketed plant and plant extract that have immunomodulatory activities.

## 2. Methods

In this review, scientific journals were selected manually on the Internet using the keywords immunomodulators plant, immunostimulant drug, immunostimulant plant, immunosuppressant drug, and plant as immunomodulators from Scopus, PubMed, Google Scholar, and ScienceDirect.

Hence, 67 journals were collected. Out of the 67 journals, 42 were referred to in addition to the six books and one website of Johns Hopkins University known with pandemic COVID-19 information.

In addition, microbiological professionals were questioned to gain insight more in detail about immunity. To draw the chemical structure of immunomodulatory compounds, ChemDraw version 8 was also used, while EndNote version 6 is used for referencing the Natural Products Database for Africa (NDA) used for botanical name cross-checking. Microsoft Excel is also used for drawing the pie chart.

## 3. Immunity System

**Figure 2:**
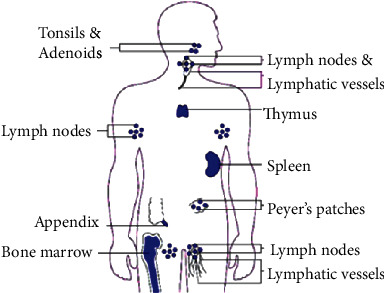
Organs of the human immune system. Adopted from [26].

It is a human defense system from endogenous and exogenous chemical and biotic invaders through the physical barrier and robotic electric or molecular reaction communication of specialized cells and organs. The biotic “foreign” invaders are called antigens (microbes—infection-causing organisms such as bacteria, viruses, parasites, and fungi or any injury- and disease-causing agent) [25]. The different organs of the human immune system are shown in Figure 2. The organs include the lymphoid, thymus, and bone marrow. The reason to put this figure is to appreciate our creator, the almighty God. If you look at the diagram, the immune system is located at the sensitive positions, accessible for pathogens to enter our body.

When an antigen makes first contact with the cell, the immune system's first action is scanning and recognizing it as self from non-self-substances [27]. Due to cell recognition, the immune system cell recognizes the self-substance as a substance belonging to the host organism. At the same time, non-self-molecules are foreign that do not belong to the host organism. One example of non-self-molecules is antigens that cause the immune system to promote the generation of antibodies against it and then combine specifically with them to induce an immune response. The generation of antibodies is due to cytokine chemicals released from the antigen-presenting cell [28]. Autoimmunity is an immune response opposed to healthy cells and tissues, leading to autoimmune diseases [29].

## 4. Type of Immunity and Immunity-Related Disorder

**Figure 3:**
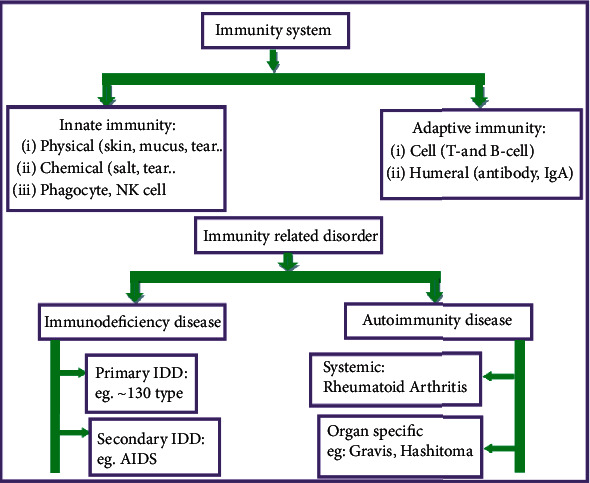
Graphical classification of the immune system and immunity-related disorder.

Human defense against infection has two primary forms, namely, innate immunity and adaptive immunity (Figure 3). Innate immunity has been considered the first line of defense against pathogens. Innate immunity includes physical barrier (hair, mucus, and skin), chemical substance (salt in the skin and tear in the eye), blood protein, phagocyte cells (macrophages and dendritic cells (DCS), mast cells, neutrophils, basophils, eosinophils, invariant natural killer cells (NK cells, NKT cells, and gd T cells). When innate immune cells and normal cells are combating pathogens, they release a chemical called cytokines. These chemicals activate adaptive immunity. Adaptive immunity then produces antibodies [18].

Adaptive immunity is mediated by cell (T and B cells) and humeral (macromolecule-like antibodies in plasm cells of blood and immunoglobulin). It employs diverse antigen receptors that are not encoded in germline cells but rather de novo generated through DNA rearrangement mechanisms in the somatic immune tissues of mammalian organisms [18].

Immunity-related disorders are of two types: immunodeficiency and autoimmune disease (Figure 3). An immune response opposed to its healthy cells and tissues is called autoimmunity, leading to autoimmune diseases [29]. Some autoimmune diseases are rheumatoid arthritis, plaque psoriasis, and some inflammatory disease. Immunocompromised diseases are of two types: primary and secondary immunodeficiency diseases. A genetic defect of immune cells causes primary immunodeficiency disease (PIDD). There are around 130 types [22]. Secondary immunodeficiency diseases are caused by diseases or environmental factors, such as malnutrition, HIV, and medical treatment (e.g., chemotherapy). The most known secondary immunodeficiency diseases are AIDS, cancer, SARS, and COVID-19 [17, 20].

The mechanism of action of macrophages is degrading the pathogens with lysosome enzymes and peroxides or nitrogen monooxide. The enzyme breaks the cell wall and attacks the nucleus and ribosome, where protein synthesis occurs [30]. The mechanism of action of the antibody is either blocking the antigen from binding to its target, labeling a pathogen for destruction by macrophages or neutrophils, or activating the complement cascade [17].

## 5. Immunostimulant Drug and Plant Bioactive Molecules

Even nature is complete with bidirectional mutual interaction. Eventually, nature also needs improvement. One of the improvements is boosting the human immune system to fight disease. Humans rely on synthetic drugs and natural plant origin immunomodulators to do so.

**Figure 4:**
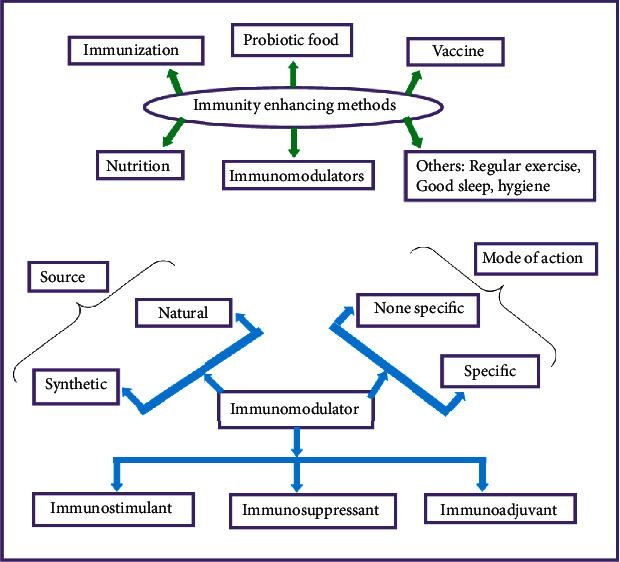
Classification of immunomodulators and immunity-enhancing methods.

Many substances that assist immunity progress are termed immunomodulators [31]. In other words, the biomolecules of biological origin or synthetic, capable of modulating, normalizing, suppressing, stimulating, or modifying any components of adaptive or innate immunity, decreasing the inflammatory responses through oxidation or alkylation are termed immunomodulators. Immunomodulators in clinical practice are mainly classified into immunosuppressants, immunostimulants, and immunoadjuvants (Figure 4). Immunoadjuvants are distinct immune mechanism stimulators added to vaccines to enhance the immune system. Immunostimulants enhance the immune system, while immunosuppressants reduce the immune system's efficacy [18, 20]. Therefore, in this section, synthetic immunomodulators with their limitations are discussed parallel to plant-derived bioactive immunomodulators. Tables 1–3 show drugs and plant-derived drugs used as immunomodulators.

Immunostimulant drugs are drugs or plant-derived bioactives that enhance the immune system's efficacy with different modes of action, mostly oxidation reactions.  Table 4 shows some immunostimulant drugs and plant-derived bioactive immunostimulants with their mechanism or mode of action.

**Figure 5:**
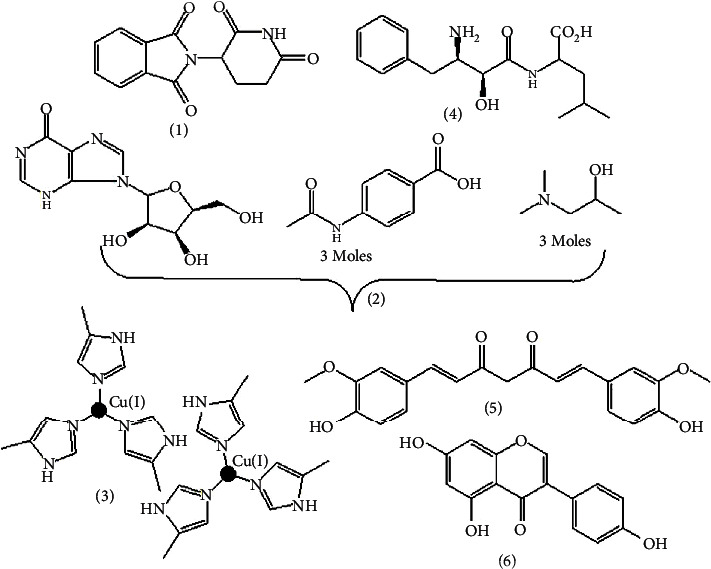
Immunostimulant drug and plant-derived bioactive immunostimulant [20].

There is also a so-called avridine, fluroquinone, polyribonucleotide immunostimulant drug. For the clinical purposes in addition to immunostimulant drugs or plant-derived drugs in Figure 5 and listed in Table 4, physicians use bacterial vaccines as immunization, carbohydrate (glucans, schizophyllan, scleroglucan, lentinan, station, bestatin, and acemannan), complimentary copolymer (polycytidylic acid), colony stimulant factors (glycoprotein stimulates WBC production, e.g., IL-3), interferons (soluble glycoprotein produced by host cell, e.g., IFNs-ἀ&*β* and IFNs-*γ*), interleukins, therapeutic and combination vaccine (e.g., Comvax and Twinrix), viral vaccine (Afluria), animal extracts (chitosan from shrimp), and plant extract (lectins and mitogens (phytohemagglutinin and concanavalin A)) as immunostimulant [16, 37].

The limitation of thalidomide (**1**) is that its S-form causes a birth defect. Isoprinosine (**2**) is a combination of inosine, acetamidobenzoic acid, and dimethylaminoisopropanol. Its disadvantages are minor CNS depressant, transient nausea, and an increased level of uric acid in serum and urine. Immunocynin (**3**) is a stable form of hemocyanin, a copper-containing protein found in mollusks and arthropods. It has poor drawbacks such as rare-mild fever. Bestatin (**4**) is an immunostimulant with low toxicity. It is a leucine aminopeptidase and aminopeptidase-B inhibitor [37].

## 6. Immunosuppressant Drug and Plant Bioactive Molecules

**Figure 6:**
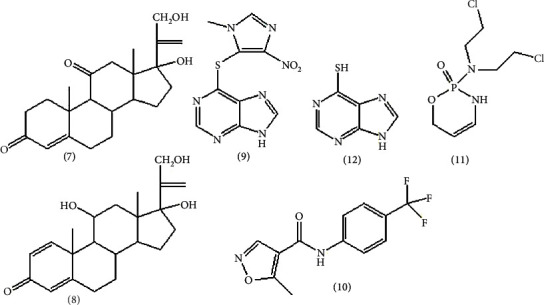
Immunosuppressant drug [29, 34].

**Figure 7:**
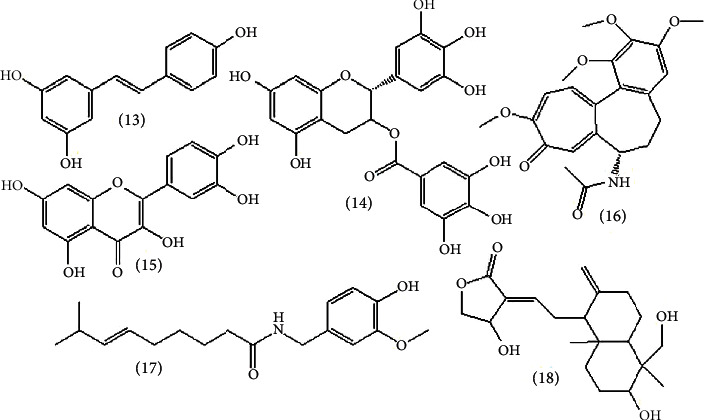
Plant-derived bioactive immunosuppressant [20, 31].

**Figure 8:**
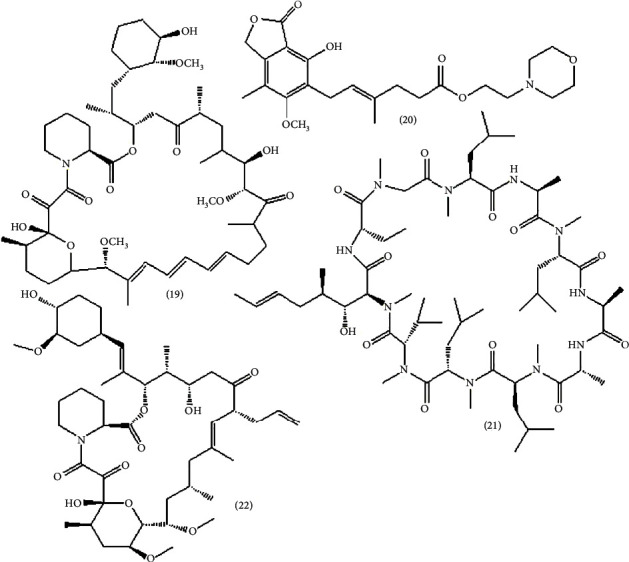
Microorganism-derived immunosuppressant bioactive drug [20, 31].

Immunosuppressants are drug that block the immune response and can be used to regulate the pathological immune response involved in organ transplants (prevent the graft-destructive immune response). Furthermore, these compounds can be used to alleviate diseases related to autoimmune disorders, hypersensitivity reactions (HR), and immunopathology (IP) or diseases originating from an autoimmune disorder [20].  Table 1 shows immunosuppressant drugs, plant-derived and microorganism-derived bioactive immunosuppressants with their use and mode of action. The structures are depicted in Figures 6–8.

Azathioprine (**9**), an immunosuppressant agent, has the following side effects: medullar suppression, myalgia, pancreatitis, hepatitis, and dizziness [32]. The limitation of cyclosporine is its narrow therapeutic index. While low concentrations in the blood cause immunosuppression, high concentrations also cause kidney malfunction [30].

## 7. Immunoadjuvant Drug and Plant Bioactive Molecules

Immunoadjuvant is a chemical combined with an antigen for increasing immune response by stabilizing the antigen (Table 2). Immunoadjuvants enhance the efficacy of vaccines. For example, Freud's adjuvant is composed of inactivated and dried mycobacterium emulsified in mineral oil [20, 37].

## 8. Plant-Derived Natural Products as Immunomodulators

Medicinal plant extract as a food supplement and medicine is now not debatable. Even the US Food and Drug Administration (FDA) approved 1453 new chemical entities from natural products in 2013. In addition, WHO listed twenty-one thousand medicinal plants worldwide [8, 32]. The reason to rely on medicinal plant extract is more negligible side effects, less cost, and a multicomponent agent as opposed to the synthetic drug [10, 11]. Medicinal plants are used as antibacterial, anti-inflammatory, antioxidant, immunomodulators, antifungal, anthelmintic, and anticancer and to treat cardiovascular disease. Even the other era of natural product chemistry is hyphenating natural products with synthetic products. This combination effect is now a new research area in natural product chemistry and chemistry as a whole. Similarly, measuring the impact of mixed plant extract is on the way [32]. The other reason why natural product chemists should develop their full potential is because there are about 400,000 plant species in the world [20]. However, only 21,000 medicinal plants are recognized by the WHO [8].

### 8.1. Immunomodulator Plant

**Figure 9:**
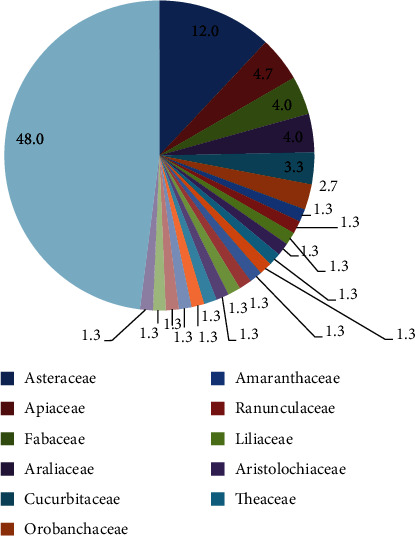
The distribution of plants by the family that has immunomodulatory activity.

Immunomodulators are compounds that catalyze immune reactions [36]. The phytochemicals used as plant-derived immunostimulant drugs are polyphenols, flavonoids, diterpenoids, alkaloids, and phytoestrogens [20, 31]. Similarly, other phytochemicals found in plants have immunomodulatory activity but have not been approved yet. This includes pentacyclic triterpenes from *Euphorbia microsciadia* [32], thymoquinone from *Nigella sativa* [40], and triterpenoid from *Scoparia dulcis* Linn. [36]. On the other hand, for example, a study was done in 2000 in Iran; four medicinal plants, *Silybum marianum*, *Matricaria chamomilla*, *Calendula officinalis*, and *Cichorium intybus*, show immunomodulatory activity on their mitogenic activity though the study does not show the responsible phytochemical [41]. Like the Iranian study, there are many immunomodulatory activity studies done worldwide. Hence according to [20, 31, 42], there are 150 medicinal plants identified to have immunomodulatory activity. In the reference mentioned above, the plant part, responsible phytochemical, and mechanism of action are tabulated in detail. However, here, the pie chart (Figure 9) is extracted and constructed from the above references, and it shows the distribution of plants by a family with immunomodulatory activity.

*Asteraceae* accounts for 12% and is put in the first rank by offering 18 plant species. Similarly, *Apiaceae* (4.7%, 7 plants), *Fabaceae* (4%, 6 plants), *Araliaceae* (4%, 6 plants), *Cucurbitaceae* (3.3%, 5 plants), and *Orobanchaceae* (2.7%, 4 plants) take second up to sixth rank with their percent and plant offer shown in parenthesis. The rest of the families that give 1.3% offer two plants each from 16 families. The others which give 48% are only one family and give only one plant (72 plants). Similarly, generally from the plants studied so far regarding immunomodulatory activity, 40% of the plant species belong to the *Asteraceae* family [38].

As different scholars classify natural products, phytochemicals as an immunomodulator can be classified into two: low and high molecular weights. Moreover, Table 3 depicts this classification [20, 38]. The high molecular weight immunomodulator natural products are not presented in detail; however, some are mentioned as carbohydrate and plant extract proteins used as immunostimulants in Section 8.1 of this paper. Glucans and iridoid glycoside are two known examples of high molecular weight polysaccharides having immunomodulatory function. The other protein found in the plant is lectins. Lectins are carbohydrate-binding proteins [38].

Besides taking immunomodulators before and after infections occur, it is also good to follow the immunity-enhancing methods described in Figure 4 to be healthier. There are a total of eight immunity-enhancing strategies. Here, it should be clear that for clinical purposes, the immunity-enhancing method is three in type, while the general methods that every healthy man should follow are eight methods, including the clinical immunity-enhancing method (Figure 3). To get more insight into immunity-enhancing methods, refer to reference [16].

## 9. Mechanism of Action of Plant Phytochemicals as Immunomodulators

**Figure 10:**
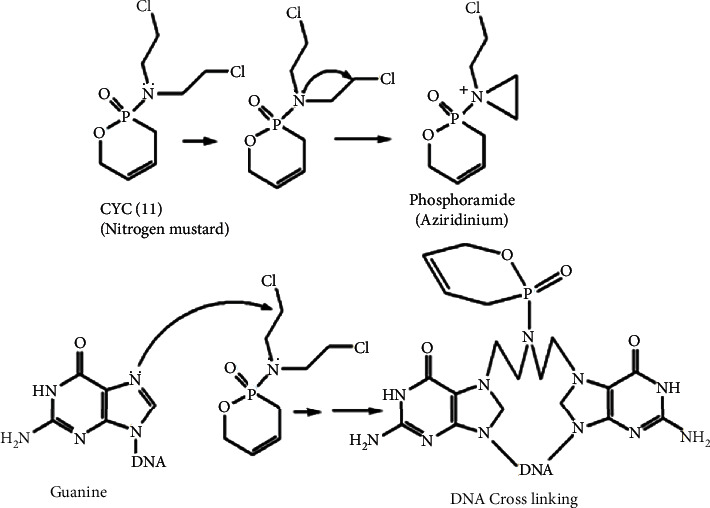
Alkylation of DNA by nitrogen mustard (e.g., CYC).

Figuratively, it is possible to categorize the mechanism or mode of action of the phytochemicals based on the method applied to measure their efficacy as *in vitro* and *in vivo* assays. *In vivo assay* is based on protecting host cells against pathogens while *in vitro assay* is of cellular and humoral immune molecular mechanisms. Therefore, the review's tool is based on *in vitro* and *in vivo* assays but does not detail pharmacodynamics and pharmacokinetics. In other words, immunomodulatory drug activity is not discussed in detail. Instead, it is more about plant-derived bioactive immunomodulators. However, as a sample, cyclophosphamide (CYC) (**11**) is one of the immunosuppressant drug modes of action due to its alkylating agent ability of DNA. CYC is a nitrogen mustard. Phosphoramide mustard is an active form of CYC that inhibits protein synthesis by alkylating DNA and cross-linking DNA at guanine N-7 sites (Figure 10). Indirectly, it kills resting and dividing lymphocytes. In a clinical test done on rheumatoid arthritis patients, the number of T cells and B cells decreased [34, 43, 44].

The mechanism or mode of action based on target specificity is of two types, specific and nonspecific, in immunomodulator medicines or plant bioactives. Specific immunomodulator types are like vaccination which works for a specific antigen. In contrast, a nonspecific immunomodulator is not designed for specific antigens but rather activates both innate immunity and adaptive immunity (Figure 4) [20].

**Figure 11:**
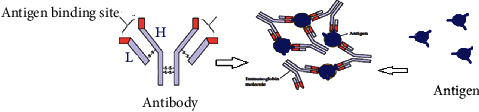
Antibody immunoglobulin and antigen interaction with cell-cell recognition (H and L are heavy and light polypeptide chains, respectively).

Plant bioactive immunomodulators' mechanism of action is generally based on activating and producing signal molecules (cytokines) of water-soluble glycoproteins. This includes colony-stimulating factors, interleukins, and interferon, produced by the host cell and can be obtain as vaccines and medicines [45]. These signal-sending molecules then activate the innate immune system (natural killer cell and macrophages) and adaptive immune system (cellular: antibody generation Ig and humoral: T and B cells). The immune system acts on antigen based on Toll-like receptor (TLR) protein and antigen interactions.  Figure 11 shows a straightforward interaction of antibody, immunoglobulin, and antigen interaction with cell-cell recognition.

So far in the discussion, one thing should be clear: immunomodulators are not anti-infective drugs or plant bioactive compounds. Instead, they increase the efficacy of anti-infective medicines like antibiotics by adding extra support from the host cell immune system. Of course, since some autoimmune diseases suppress the immune system, they can be considered anti-infective medicines. For example, for a disease called rheumatoid arthritis type which affects 0.6% of the US population [21], immunosuppressive drugs cortisone [30] and azathioprine [32] and natural product plant extract can be applied.

With the above idea in mind and referring to the low molecular weight class of chemicals, phenolic compounds, terpenoids, and alkaloids, the immunomodulatory mechanism of action is discussed by taking one specific compound from each class. Since structure-activity relations between different and within-class of a natural product are not often clear, it makes it difficult to classify the mechanism. However, induction of cytokines, the release of iNOS, and enhancement of phagocytosis are the most general mechanism shown by low molecular weight immunomodulator phytochemicals. Even though the mechanisms of the high molecular weight phytochemicals are limited to discuss here in this review, generally they exert their effect through stimulating macrophage function.

### 9.1. Phenolic and Flavonoid Compound, Resveratrol

**Figure 12:**
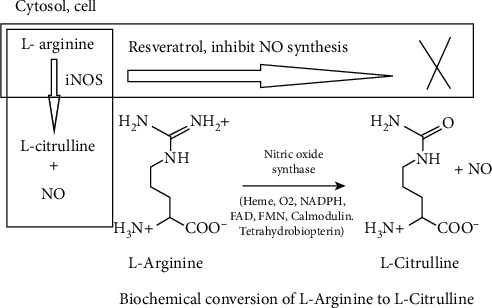
iNOS inhibition by resveratrol in the cytosol of the cell.

Resveratrol (**13**) is derived from stilbene and phytoalexin. Grapevines, red wine, and peanuts are the main sources. The immunostimulant activities of resveratrol are due to the inhibition of the generation of iNOS in LPS- (lipopolysaccharide-) activated macrophages [38]. The enzyme nitric oxide synthases (NOS) catalyze the transformation of L-arginine to L-citrulline and nitric oxide (NO). This conversion occurs in the cytosol of cells (Figure 12). NO works as a cellular signaling molecule in response to cytokines and also attacks parasites, bacterial infection, and tumor growth since it is radical [38]. However, it is important to reduce NO production especially for cancer treatment in some cases. Chemically inducible NOS (iNOS) is a protein [46].

**Figure 13:**
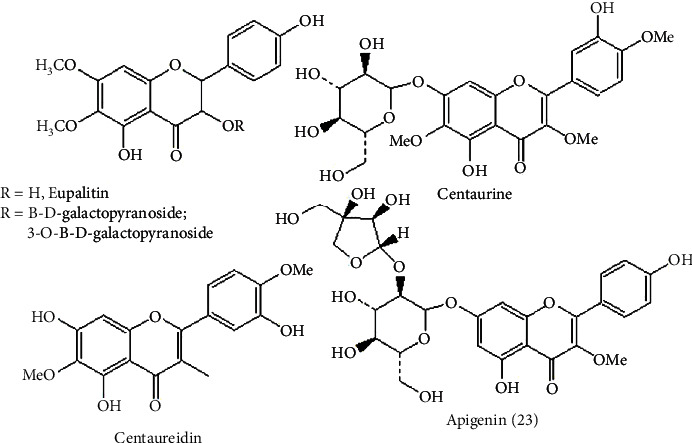
Flavonoid immunomodulator.

In addition, according to [13], there are different flavonoids including apigenin (**23**), as shown in Figure 13, isolated from *Terminalia arjuna* plants that have immunomodulatory activity [13].

### 9.2. Alkaloids

**Figure 14:**
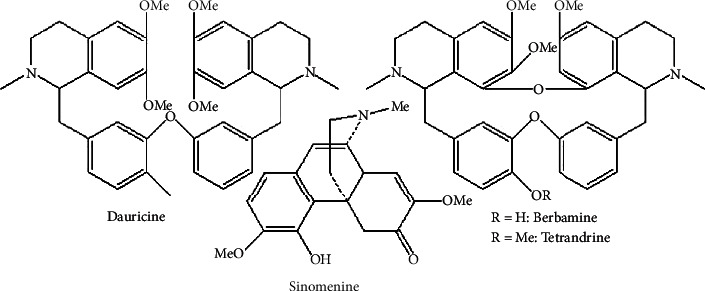
Alkaloid immunomodulator.

The two coca plant species *Erythroxylum coca* and *Erythroxylum novogranatense* are sources of a known alkaloid called cocaine. It affects the immune system by activating macrophage and splenocyte function by releasing IL-1*a*, IL-6, and TNF-*a*; splenocytes also release IFN-*γ*, IL-2, IL-4, IL-5, and IL-10 [38]. In addition, Figure 14 shows alkaloids that have immunomodulatory activity [13].

### 9.3. Terpenoids, Andrographolide

**Figure 15:**
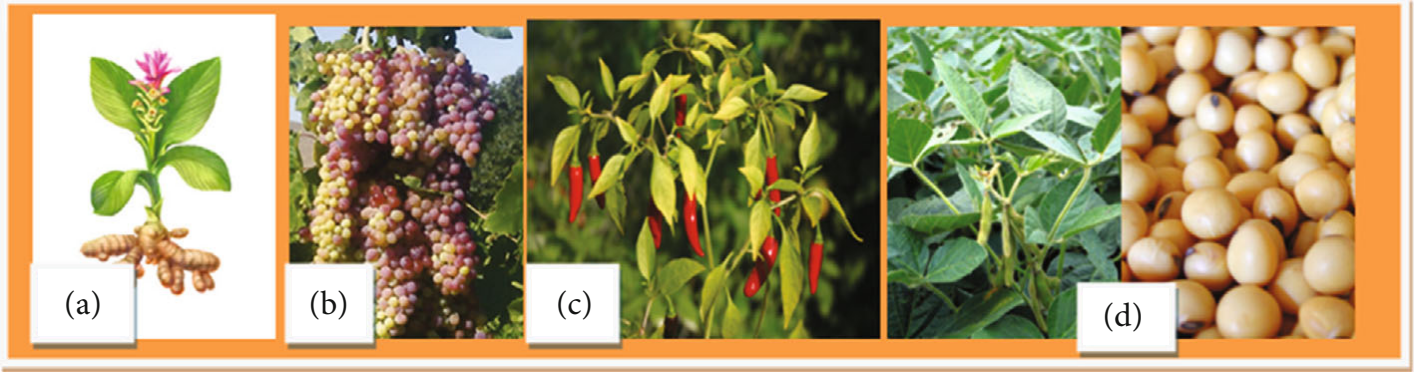
Immunomodulator of cultivated medicinal plants. (a) Turmeric. (b) Grape. (c) Chile paper. (d) Soybean.

The known diterpenoid found in the plant *Andrographis paniculata* is called andrographolide (**18**) (Figure 7). Its immunomodulatory activities have been observed *in vitro*, including the reduction of IL-12, TNF-*α*, PGE2, NO, COX-2, and iNOS in microglia and macrophages. Macrophage activity expressed by iNOS and COX-2 through LPS stimulation was inhibited by andrographolide [20, 31].  Figure 15 shows plants that are discussed in Sections 6 and 8.

## 10. Clinical Trials of Plant-Derived Molecules

**Figure 16:**
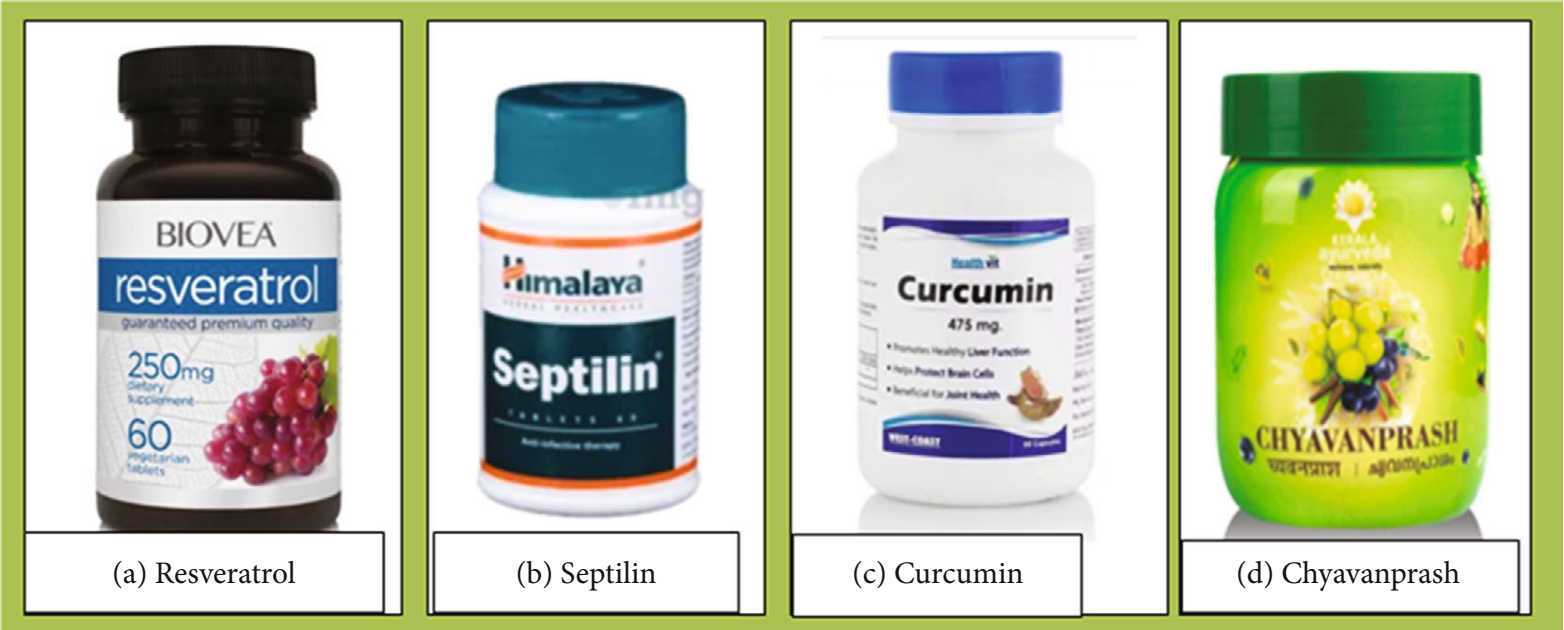
Medicinal plant extract sold in the market claimed to have immunomodulatory activity.

As history tells, the use of immunostimulation therapy started in 1857 by Ferdinand Hoff by injecting milk or blood protein to treat malaria and lues [38]. Hence, the eight plant-derived immunomodulator bioactive compounds discussed in Section 4 of this review have already passed the clinical test and are approved. There are two immunostimulants, Curcumin (*Curcuma longa*) and Genistein (soy), and six immunosuppressants, resveratrol (grapevines, red wine, and peanuts), epigallocatechin-3-gallate (green tea *Camellia sinensis*), quercetin (tea, capers, red onions, broccoli, berries, grapevines, and apples), colchicine (*Colchicum autumnale* (family: *Colchicaceae*)), capsaicin (chili peppers (*Capsicum* species; *Solanaceae*)), and andrographolide (*Andrographis paniculata)* [31]. However, more work is still undergoing to get more immunomodulatory agents. For example, a combination of four medicinal plants by [47] was studied against immunomodulatory activity using *in vivo* test of cyclophosphamide-induced immunosuppressed mice. They found that this polyherbal medicine is effective. Figuratively, the polyherbal significantly (*p* < 0.01) enhanced the immune cells like natural killer (NK) cells, B cells, CD4 cells, and CD8 cells by 60%, 18%, 14%, and CD8 7%, respectively. Hence, several polyherbal medicines are found in the market, claiming to be immunomodulators. Vivartana, Chyawanprash, Brahma Rasayana, IM-133, and Septilin are some to mention that are formulated from the Indian traditional system of medicine, and they are clinically approved (Figure 16) [47].

Septilin is a Hindu traditionally formulated medicine produced in India. It is a polyherbal drug. A preclinical test on Septilin by cyclophosphamide-induced immunosuppressed mice confirms its immunomodulatory activity [48]. Previously, these researchers [49] also did the cytotoxicity of Septilin against cisplatin- (Csp-) induced human breast adenocarcinoma (MCF-7) and normal human breast epithelial (MCF-10A) cell lines. They found that the polyherbal drug, Septilin, does not exert cytotoxicity on the normal human breast epithelial (MCF-10A) cell while it exerts its cytotoxicity on cisplatin-induced human breast adenocarcinoma (MCF-7) with a concentration of 5 *μ*g/mL. This indicates that the polyherbal drug Septilin is safe to be used as a drug like the other plant source immunomodulatory drugs or phytochemicals.

**Figure 17:**
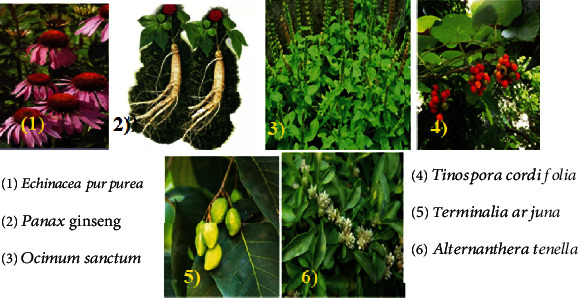
A list of immunomodulator medicinal plant.

In addition to polyphenols, terpenoids, and alkaloids, saponins are good immunostimulants. For example, cycloartane and oleanane-type unmistakably induced interleukin-2 activity [13].  Figure 17 shows some medicinal plant lists and images of the plant that have immunostimulant activity (1-5) and immunosuppressant activity (6).

## 11. Conclusion

Several plants' phytochemicals have been identified over the years for their immunomodulatory activity. Instead of commercial drugs, numerous illnesses can be alternatively treated by immunomodulation using medicinal plants. The discovery and isolation of more specific immunomodulatory agents from plant origin possess the potential to counteract the side effects and high cost of synthetic compounds. Taking plants and plant-derived immunomodulators, such as resveratrol meal supplements available from a pharmacy, has been popular in several nations recently. Many plant families are reported to have immunomodulatory activity; the *Asteraceae* family is an important one. *Echinacea purpurea* of this family is most known for its immunostimulating activity. Phenolic carboxylic acids, terpenoids, and alkaloids are the most prominently reported immune-active bioactive molecules. There are eight plant bioactive immunomodulators checked for clinical trials and found in the market. These are six immunosuppressants, resveratrol, epigallocatechin-3-gallate, quercetin, colchicine, capsaicin, andrographolide, and two immunostimulants, curcumin and genistein. This suggests that since our world is replete with therapeutic plants, considerable research into plant-derived immunomodulators will be necessary in the future to fully understand their mechanisms of action at the systemic, cellular, and molecular levels and extension to a broad-spectrum clinical trial.

## Figures and Tables

**Table 1:** Immunosuppressant drug, plant-derived bioactive, and microbe-derived drugs.

Drugs	Use	Mode of action	Ref.
*Immunosuppressant*			
(7) Cortisone and/or (8) prednisolone	To treat rheumatoid arthritis disease (autoimmune disease)	Decreased leukocyte migration to sites of inflammation	[30]
(9) Azathioprine	Used in rheumatoid arthritis, granulomatosis with polyangiitis, Crohn's disease, ulcerative colitis, and antirejection	Inhibits purine synthesis and less DNA and RNA are produced for the synthesis of white blood cells, thus causing immunosuppression	[32]
(10) Leflunomide	Antirejection	Suppress T and B cell proliferations	[33, 34]
(11) Cyclophosphamide	Treat malignancies	Inhibit T and B cell proliferations	[35]
(12) 6-Marcaptopurine	Antiviral and anticancer	Interferon inducer	[36]

*Plant-derived bioactive*			
(13) Resveratrol (grape) and (14) epigallocatechin-3-gallate (green tea) (polyphenols)	Anti-inflammatory, anticancer	By blocking NF-*κ*B in LPS and PMA and blocking COX-2	[20, 31]
(15) Quercetin (grapes) (flavonoids of polyphenol)	Antimutagenic, neuroprotective, antioxidative, anti-inflammatory, and anticancer	Inhibits the action of COX-2 by suppressing NF-*κ*B, AP-1, and STAT-1 signaling in cytokines or LPS-activated macrophages	[20, 31]
(16) Colchicine and (17) capsaicin (chill paper) (both are alkaloid)	Treat the familial Mediterranean fever and acute gout flares (FAD approved)	Inhibits activation and migration of neutrophils to sites of inflammation	[20, 31]
(18) Andrographolide (diterpenoid)	Treat cancer	Inhibits cancer cells growth by immunomodulatory effect and anti-inflammatory	[20, 31]

*Microbe-derived drug*			
(19) Sirolimus (from bacteria)	Antirejection	Inhibits T lymphocyte activation and proliferation	[33, 34]
(20) Mycophenolate mofetil (from bacteria)	Antitumor	Inhibit IMDH	[33, 34]
(21) Cyclosporine (from soil fungus) (glycoprotein)	Antirejection	Suppress the production of immunoglobulins against foreign proteins in the new organ	[30]
(22) Tacrolimus (from bacteria)	Antirejection	Inhibit lymphocyte inhibition	[33, 34]

**Table 2:** Immunoadjuvant drug and plant-derived bioactive immunoadjuvant.

Drugs	Use	Mode of action	Ref.
*Immunoadjuvant*			
(1) Levamisole (Ergamisol)	Adjuvant therapy with 5-fluorouracil after surgical resection in patients with Duke's stage C colon cancer	Inducing B and T lymphocytes, monocytes, and macrophages	[37]

*Plant-derived bioactive*			
(1) Pectic arabinogalactan (*Cistanche deserticola*) (polysaccharide)	Enhancing immune response of thymus gland by combining with 3-(4,5-dimethylthiazol-2-yl)-2,5-diphenyltetrazolium bromide	Enhancing effect of polysaccharides on murine thymus lymphocyte proliferation was related to its delivery of thymus intracellular calcium ions	[13]

**Table 3:** Low and high molecular weight natural products used as an immunomodulator.

	Example	Reference
Low molecular weight compounds		
(1) Alkaloid	Cocaine, vincristine	[20, 38]
(2) Terpenoids	Sesquiterpene lactones, diterpenes, and triterpenes	[20, 38]
(3) Phenolics	Flavonoids, coumarins, and quinones	[20, 38]

High molecular weight compounds		
(1) Lectins	Concanavalin A	[20, 37, 38]
(2) Polysaccharides	Glucans, lentinans, and arabinogalactans	[20, 38]

**Table 4:** Immunostimulant drug and plant-derived bioactive immunostimulant.

Drugs	Use	Mode of action	Ref.
*Immunostimulant*			
(1) (R)-Thalidomide or lenalidomide and pomalidomide	Treat multiple myeloma (cancer of plasma cell), treat rheumatoid arthritis and angiogenesis	Inhibit myeloma proliferation that reduces antibody production	[16, 37]
(2) Isoprinosine (inosiplex/Imunovir)	Treat herpes simplex infections, Epstein-Barr, and measles viruses	Enhance the levels of cytokines (IL-1, IL-2, and IFN-*γ*), a proliferation of lymphocytes, augmented active T cells, and induced T cell surface markers on prothymocytes	[37]
(3) Immunocynin (hemocyanin)	Treat urinary bladder cancer	Carry oxygen to the affected area	[37]
(4) Bestatin	Antitumor activity and also increase the antitumor activity of bleomycin and adriamycin	Binds to the cell surface of lymphocytes and macrophages and enhances adaptive immune responses	[37]

*Plant bioactive*			
(5) Curcumin (turmeric) (polyphenol)	Antiproliferative, anticancer, proapoptotic, antiangiogenic, and antioxidant	Increase WBC count	[20, 39]
(6) Genistein (soy) (phytoestrogen)	Treat diabetes	Produce NO and PGE2, increase insulin resistance	[20, 31]
